# Experimental blunt chest trauma – cardiorespiratory effects of different mechanical ventilation strategies with high positive end-expiratory pressure: a randomized controlled study

**DOI:** 10.1186/s12871-015-0166-x

**Published:** 2016-01-12

**Authors:** Dierk Schreiter, Nadja C. Carvalho, Sebastian Katscher, Ludger Mende, Alexander P. Reske, Peter M. Spieth, Alysson R. Carvalho, Alessandro Beda, Burkhard Lachmann, Marcelo B. P. Amato, Hermann Wrigge, Andreas W. Reske

**Affiliations:** 1 Helios Park Clinic, Department of Intensive Care Medicine, Leipzig, Germany; 2 Department of Anesthesiology and Intensive Care Medicine, University Hospital Leipzig, Liebigstr. 20, D-04103 Leipzig, Germany; 3 Department of Electronic Engineering, Federal University of Minas Gerais, Belo Horizonte, Brazil; 4 Sana Kliniken Leipziger Land, Department of Orthopedic, Trauma and Hand Surgery, Borna, Germany; 5 Intensive Care Unit, Sana Kliniken Leipziger Land, Borna, Germany; 6 Anesthesiology and Intensive Care Medicine, Fachkrankenhaus Coswig, Coswig, Sachsen Germany; 7 Pulmonary Engineering Group, Department of Anesthesiology and Intensive Care Medicine, University Hospital Carl Gustav Carus, Dresden, Germany; 8 Carlos Chagas Biophysics Institute, Federal University of Rio de Janeiro, Rio de Janeiro, Brazil; 9 Department of Anesthesiology and Intensive Care Medicine, Charité, Berlin Medical University, Berlin, Germany; 10 Cardio-Pulmonary Department, Pulmonary Division, Hospital das Clínicas, University of São Paulo, São Paulo, Brazil

**Keywords:** Pulmonary contusion, Blunt chest trauma, Acute respiratory distress syndrome, Mechanical ventilation, Positive end-expiratory pressure, Hypercapnia, Computed tomography

## Abstract

**Background:**

Uncertainty persists regarding the optimal ventilatory strategy in trauma patients developing acute respiratory distress syndrome (ARDS). This work aims to assess the effects of two mechanical ventilation strategies with high positive end-expiratory pressure (PEEP) in experimental ARDS following blunt chest trauma.

**Methods:**

Twenty-six juvenile pigs were anesthetized, tracheotomized and mechanically ventilated. A contusion was applied to the right chest using a bolt-shot device. Ninety minutes after contusion, animals were randomized to two different ventilation modes, applied for 24 h: Twelve pigs received conventional pressure-controlled ventilation with moderately low tidal volumes (V_T_, 8 ml/kg) and empirically chosen high external PEEP (16cmH_2_O) and are referred to as the HP-CMV-group. The other group (*n* = 14) underwent high-frequency inverse-ratio pressure-controlled ventilation (HFPPV) involving respiratory rate of 65breaths · min^−1^, inspiratory-to-expiratory-ratio 2:1, development of intrinsic PEEP and recruitment maneuvers, compatible with the rationale of the Open Lung Concept. Hemodynamics, gas exchange and respiratory mechanics were monitored during 24 h. Computed tomography and histology were analyzed in subgroups.

**Results:**

Comparing changes which occurred from randomization (90 min after chest trauma) over the 24-h treatment period, groups differed statistically significantly (all P values for group effect <0.001, General Linear Model analysis) for the following parameters (values are mean ± SD for randomization vs. 24-h): PaO_2_ (100 % O_2_) (HFPPV 186 ± 82 vs. 450 ± 59 mmHg; HP-CMV 249 ± 73 vs. 243 ± 81 mmHg), venous admixture (HFPPV 34 ± 9.8 vs. 11.2 ± 3.7 %; HP-CMV 33.9 ± 10.5 vs. 21.8 ± 7.2 %), PaCO_2_ (HFPPV 46.9 ± 6.8 vs. 33.1 ± 2.4 mmHg; HP-CMV 46.3 ± 11.9 vs. 59.7 ± 18.3 mmHg) and normally aerated lung mass (HFPPV 42.8 ± 11.8 vs. 74.6 ± 10.0 %; HP-CMV 40.7 ± 8.6 vs. 53.4 ± 11.6 %). Improvements occurring after recruitment in the HFPPV-group persisted throughout the study. Peak airway pressure and V_T_ did not differ significantly. HFPPV animals had lower atelectasis and inflammation scores in gravity-dependent lung areas.

**Conclusions:**

In this model of ARDS following unilateral blunt chest trauma, HFPPV ventilation improved respiratory function and fulfilled relevant ventilation endpoints for trauma patients, i.e. restoration of oxygenation and lung aeration while avoiding hypercapnia and respiratory acidosis.

## Background

Current concepts for ventilatory support in patients suffering from acute respiratory distress syndrome (ARDS) aim to reduce ventilator-associated lung injury by limiting tidal volumes (V_T_) and airway pressures. Tolerance of side effects such as low arterial oxygenation levels and hypercapnia is part of lung protective ventilation strategies in general ARDS populations [1–3]. In trauma-associated ARDS however, such permissive ventilator management may conflict with the treatment of traumatized patients [4, 5]. The risk of acute bleeding events or tissue hypoxia, for example, may prompt physicians to secure normal oxygenation and oxygen contents [5–7], but common therapeutic options such as ventilation with higher positive end-expiratory pressure (PEEP), prone positioning or early spontaneous breathing, may be limited in trauma patients with severe unilateral lung injury, severe pain, instable pelvic or spine fractures or severe brain injury [8–10]. Moreover, the avoidance of hypercapnic acidosis in patients with brain trauma or impaired coagulation may lead physicians to refrain from using low V_T_ [3, 4, 9, 11–13].

Consequently, a dilemma is often faced when trauma patients develop ARDS and uncertainty persists regarding the optimal ventilatory strategy [13–17]. This problem particularly affects patients who sustained blunt chest trauma, which is frequently associated with both early posttraumatic ARDS and concomitant severe brain trauma [13, 18]. In this context, the rationale of the Open Lung Concept, namely early lung recruitment and restoration of lung aeration and gas exchange, may be of interest [6, 17, 19–22]. While the Open Lung Concept can be applied by different modes of mechanical ventilation [6, 17, 19, 23–25], it was implemented in this work by high-frequency inverse-ratio pressure-controlled ventilation (HFPPV) [6, 19, 23]: lung recruitment was performed and total PEEP (PEEP_tot_) was increased by generation of intrinsic PEEP (PEEP_int_) in addition to external PEEP (PEEP_ext_) set on the ventilator. PEEP_int_ was generated by shortening expiratory times using an inspiratory-to-expiratory time ratio of 2:1 and high respiratory rates which have both been reported as options to improve oxygenation and/or CO_2_ elimination [6, 19, 23, 26–28].

We previously applied the HFPPV as rescue strategy in patients with posttraumatic ARDS who showed progressively worsening lung function despite mechanical ventilation with reduced V_T_ at high PEEP. The HFPPV resulted in significant improvements of lung aeration and gas exchange and allowed tight PaCO_2_ control [6]. To improve our understanding of the physiological mechanisms governing these uncontrolled clinical observations, we performed this randomized controlled experiment in pigs. We hypothesized that HFPPV fulfilling the Open Lung principles over a prolonged period of 24 h in pigs with blunt chest trauma would allow improvements in oxygenation and lung recruitment beyond those reached by conventional mechanical ventilation with moderately low V_T_ and high PEEP_ext_ (HP-CMV) while avoiding hypercapnia and acidosis associated with the latter.

## Methods

The study was approved by the animal ethics authority Ministerium für Ernährung, Landwirtschaft und Forsten des Landes Brandenburg (Potsdam, Germany, reference number 48-3560/56). Animals were handled according to the NIH principles of laboratory animal care [29].

### Anesthesia, surgical preparation and general supportive management

Animals were screened clinically for preexisting infections. After intramuscular injection of azaperone (1 mg · kg^−1^), midazolam (3 mg · kg^−1^) and ketamine (15 mg · kg^−1^), animals were tracheotomized and mechanically ventilated (8 mm endotracheal tube). Anesthesia and analgesia was maintained by infusion of ketamine (5-30 mg · kg^−1^ · h^−1^) and midazolam (1-5 mg · kg^−1^ · h^−1^). Pancuronium (0.2 mg · kg^−1^ · h^−1^) was infused continuously. Vascular catheters were inserted into the left external jugular vein, the pulmonary artery and a femoral artery by sterile surgical preparation. Ringer's lactate solution was continuously infused at a rate of 5 ml · kg^−1^ · h^−1^, which was increased if central venous pressure was below 5 mmHg, diuresis <1 ml · kg^−1^ · h^−1^, heart rate >140 min^−1^, or mean arterial pressure <65 mmHg. We did not attempt recording or analysis of infusion volumes or vasopressor doses beyond following the aforementioned standardized guidelines for volume or vasopressor therapy. Infusion rate was reduced if central venous pressure exceeded 15 mmHg or the fluid balance exceeded 100 ml · h^−1^. Hypotension persisting despite fluid replacement was treated with intravenous norepinephrine (10 μg bolus injections or continuous infusion of doses between 0.05 and 0.5 μg · kg^−1^ · min^−1^) targeted to maintain mean arterial pressure above 65 mmHg. Propranolol (0.03 mg · kg^−1^ bolus injections) was administered intravenously when arrhythmia and tachycardia developed after trauma. Body temperature was maintained using a heating blanket. All pigs received 2 g mezlocilline intravenously every 8 h. To avoid hypoglycemia, 5 % glucose solution was infused (1.5 ml · kg^−1^ · h^−1^). Animals were euthanized after the experiment by injection of 60 ml of 1 molar potassium chloride.

### General protocol, monitoring, data acquisition and supportive therapy

After 60 min stabilization, baseline measurements were obtained. Thereafter, the chest trauma was applied and all pigs were subsequently ventilated with non-individualized settings commonly applied in the prehospital rescue and transportation setting for 90 min. For standardized measurements just before randomization the F_I_O_2_ was increased to 1.0. Immediately afterwards, animals were randomized and allocated to the HFPPV or HP-CMV groups using sealed envelopes. Hemodynamics, gas exchange, and respiratory mechanics were recorded for 24 h.

Mean pulmonary artery, mean arterial, and central venous pressures as well as heart rate were continuously monitored (SC9000, Siemens, Erlangen, Germany). Cardiac output and pulmonary capillary wedge pressure were measured using the pulmonary artery catheter (Opti-Q™ and OXIMETRIX^®^ 3 system, Abbott Laboratories, Illinois, USA). Arterial blood gases were monitored continuously (Trendcare, Diametrics Medical, High Newcombe, UK). For calculations and calibration of the blood gas monitor, conventional blood gas analysis was performed (Immediate Response Mobile Analysis (IRMA), Diametrics Medical) during ventilation with the maintenance fraction of inspired oxygen (F_I_O_2_). Oxygen delivery (DO_2_), oxygen consumption (VO_2_), and venous admixture (Q_VA_/Q_T_) were calculated using standard equations [30].

Airway pressures and V_T_ were read from the ventilator’s screen (Servo300A with Servo-Screen, MAQUET Critical Care, Solna, Sweden). An end-expiratory hold was performed for measuring PEEP_int_. A heat and moisture exchanger was inserted between endotracheal tube and Y-piece.

### Application of the blunt chest trauma

According to previous reports [31–33], a lead plate (5 cm · 5 cm) was mounted on a steel plate (5 cm · 10 cm) and taped to the right chest wall in the anterior axillary line just below the axilla. After 3 min ventilation with pure oxygen and supplementary doses of ketamine (3 mg · kg^−1^) and midazolam (0.5 mg · kg^−1^), the endotracheal tube was clamped at end-inspiration just before applying the chest trauma with a bolt-shot device (Kaliber 9x17, Modell Blitz-Export, JOPP GmbH, Bad Neustadt, Germany) [31–33]. Immediately afterwards, chest tubes were inserted on both sides [34].

### Mechanical ventilation during surgical preparation and the prehospital period

During instrumentation and for 90 min after trauma, volume-controlled ventilation mimicing clinical reality with the technically simple transport ventilators and monitors was performed: V_T_ was 350 ml and PEEP 2cmH_2_O for all animals, the inflating pressure (P_high_) was limited to 35cmH_2_O. The respiratory rate was initially set to 20breaths · min^−1^. Respiratory rate and V_T_ were subsequently adjusted to maintain end-expiratory PCO_2_ between 35 and 45 mmHg. The F_I_O_2_ was adjusted to keep the peripheral oxygen saturation above 90 %. Before baseline measurements, the inflating pressure (P_high_) was increased to 40cmH_2_O for 10 breaths to minimize atelectasis, which might have developed during instrumentation [35].

### Mechanical ventilation after randomization

In both groups, mechanical ventilation was delivered using the standard ventilator (Servo300A, MAQUET) without study-specific modifications. In the HP-CMV-group, conventional pressure-controlled ventilation (PCV) was performed with V_T_ of 8 ml · kg^−1^. Because lung contusion causes inhomogeneous lung injury, an individualized selection of PEEP by methods such as adding 2 cmH_2_O to the lower inflection point of the pressure-volume-curve of the respiratory system or best dynamic compliance can be cumbersome if not impossible [1, 36–38]. Therefore, if no clear lower inflection point could be identified, we empirically chose a high PEEP_ext_ of 16cmH_2_O, which was kept unchanged throughout the study [1, 38, 39]. The P_high_ set on the ventilator was always kept below 35cmH_2_O [1, 2], even if it generated V_T_ slightly below 8 ml · kg^−1^. The inspiratory-to-expiratory time ratio was 1:1. The F_I_O_2_, which had been set to 1.0 before randomization, was subsequently adjusted to a level maintaining PaO_2_ above 60 mmHg throughout the study. The respiratory rate was initially set to 20breaths · min^−1^ and could only be increased as long as expiratory flow reached zero to exclude development of PEEP_int_. When severe hypercapnia and acidosis (PaCO_2_ > 80 mmHg and pH < 7.20) developed, P_high_ and thus V_T_ could be increased as long as P_high_ remained ≤ 35cmH_2_O [1, 2].

In the HFPPV-group, similar target settings for mechanical ventilation (i.e., PCV, P_high_ below 35cmH_2_O, and V_T_ ≤ 8 ml · kg^−1^) were used. In contrast to the HP-CMV-group, high levels of PEEP_tot_ were applied by combination of PEEP_ext_ (10cmH_2_O) and PEEP_int_, generated by high-frequency inverse-ratio PCV (inspiratory-to-expiratory time ratio 2:1 and respiratory rate 65breaths · min^−1^) [6, 19]. For recruitment, we used three predefined opening pressures (50, 65, 80cmH_2_O) in a goal-directed manner using a target-PaO_2_ > 400 mmHg (F_I_O_2_ 1.0) as indicator of sufficient alveolar recruitment [40]. We started with P_high_ of 50cmH_2_O, which was applied for approximately 10s. Afterwards P_high_ was immediately reduced to 35cmH_2_O, a safety margin also used by others [1]. If the PaO_2_ reached 400 mmHg, full recruitment was assumed [40, 41]. If not, P_high_ of 65cmH_2_O was applied. In three animals we could not achieve our target-PaO_2_ even with P_high_ of 65cmH_2_O and therefore used 80cmH_2_O. If a PaO_2_ above 400 mmHg could be reached by recruitment, but not stabilized during ventilation with P_high_ 35cmH_2_O, PEEP_ext_ was increased by 2cmH_2_O and recruitment was repeated using the previously sufficient opening pressure. If the PaO_2_ remained above 400 mmHg for 30 min during ventilation with P_high_ of 35cmH_2_O, we started to reduce P_high_. However, during high-frequency inverse-ratio PCV, P_high_ is the major determinant of PEEP_int_ provided that respiratory rate and inspiratory-to-expiratory time ratio are constant. Thus, a reduction in P_high_ reduces V_T_ and PEEP_int_ and thus PEEP_tot_. To avoid derecruitment because of too quick reduction of PEEP_tot_, the possibility of decreasing P_high_ by 2cmH_2_O was tested every hour until P_high_ was equal or below 30cmH_2_O. If PaO_2_ decreased abruptly below 400 mmHg after reduction of P_high_, derecruitment was assumed and recruitment using the previously applied P_high_ was repeated. After recruitment, ventilation was continued using the P_high_, which had been applied just before derecruitment occurred. For changing CO_2_ elimination, respiratory rate was adjusted between 60 and 80breaths · min^−1^. After each change in respiratory rate, a potential change in PEEP_int_ was excluded by measuring PEEP_tot_.

Tracheal suctioning was only performed if inevitable. After suctioning, or after accidental disconnection of the ventilator circuit, ventilation was resumed without interventions in HP-CMV-animals, while in the HFPPV-group ventilation was continued after recruitment. We did not attempt recording or analysis of the number of suctioning maneuvers or disconnections because the management of these situations inherently differs between the two strategies.

### Computed tomography

A mobile computed tomography (CT)-scanner (Tomoscan M, Philips Medical System, Hamburg, Germany) was rented and set up in our large animal research facility specifically for our experiment. Because of delayed availability of this CT-scanner, CT could be used only in a subgroup of animals (11 HP-CMV, 9 HFPPV). Single-slice CT-scans were taken at the level of the contusion during breath-hold at end-inspiratory pressure for three time points (Baseline, Randomization, 24-h). CT-scans were taken at 120 kV and 250 mAs. Images were reconstructed with 3 mm slice thickness and the SF7 (Philips’ notation) reconstruction filter. For quantitative CT analysis, the lung parenchyma was manually delineated using the Osiris software (University Hospital of Geneva, Switzerland) [41]. Differently aerated lung compartments were classified as hyperinflated (−1000 to −951 HU), normally aerated (−950 to −501 HU), poorly aerated (−500 to −101 HU) and non-aerated (−100 to +100 HU) and their size calculated as percentage of the total lung mass present in the single CT-slice [41]. We used a lower HU-threshold for defining hyperinflated and normally aerated compartments because thin CT-slices and an edge-enhancing filter were used [42].

### Histological analysis

Heart and lungs were removed *en bloc* keeping the lung inflated at the last mean airway pressure. Lung tissue (approximately 8 cm^3^) was sampled from gravity-dependent (dorsal) and central regions of the left and right lower lobe and from the non-dependent zones (ventral) of the left and right upper lobe. Following immersion in 4 % buffered formaldehyde for three days, tissue samples were embedded in paraffin, cut in 5 μm slices, stained with hematoxylin-eosin and analyzed using standard techniques [43]. A semi-quantitative score was used to assess the histological criteria atelectasis, edema, inflammation (accumulation of inflammatory cells in airspaces and interstitium), and hemorrhage by a pathologist blinded to group allocation. These characteristics were subjectively scored on a scale from 0 to 3: 0 = no presence of the feature, 1 = mild presence of the feature, 2 = moderate presence of that feature, and 3 = severe involvement [43, 44].

### Statistical analysis

Data are presented as mean (standard deviation, SD) or medians (interquartile range, IQR). For presentation of some results grand means were calculated over all measurement points after randomization. For hemodynamic and ventilatory parameters the means of triplicate measurements were entered into the study database. Histograms and D’Agostino and Pearson’s test were used to check for normal distribution. If the assumption of homogeneity of variance (Levene’s test) was violated, data were logarithmically transformed to obtain normally distributed residuals. Baseline and Randomization were compared with paired-samples t-tests or Wilcoxon’s signed-rank tests. Group effect after Randomization was tested with general linear models (GLM) adjusted for repeated measurements. The Sidak’s procedure was applied for post-hoc comparisons. Software packages SPSS 15.0 (SPSS GmbH, Munich, Germany) and GraphPad Prism 5 (GraphPad Software, La Jolla, CA, USA) were used. Significance was accepted at *P* < 0.05.

## Results

### General aspects

**Fig. 1:**
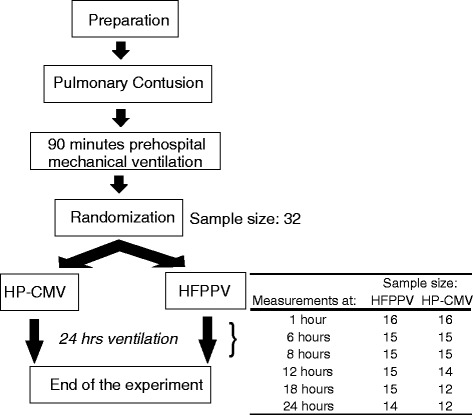
Study protocol and time course of interventions

Out of the 40 (26 female) pigs included, eight pigs died from hemorrhage, extrapulmonary injuries (liver or heart), cardiac arrhythmia or intractable shock before entering the randomized ventilation period. Of the remaining 32 pigs, two HFPPV-pigs and four HP-CMV-pigs died during randomized ventilation. Because animals were only included in the analysis if they had survived at least half of the experimental time, 26 animals (14 HFPPV- and 12 HP-CMV-pigs) were used in the analyses. The flow-chart in Fig. 1 depicts the main interventions and the number of animals remaining in the study at each measurement point.

Mean body weight did not differ between groups (HFPPV 32.4 ± 3.4 kg, HP-CMV 32.0 ± 3.8 kg, *P* = 0.7), neither did mean body length (HFPPV 104.8 ± 7.5 cm, HP-CMV 106.8 ± 7.5 cm, *P* = 0.55).

**Fig. 2:**
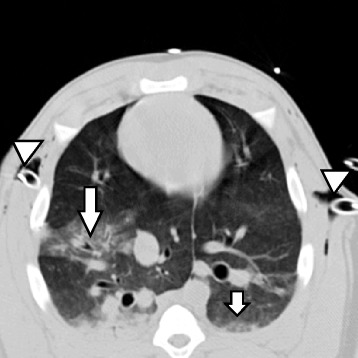
Representative CT image taken during breath-hold at end-inspiratory pressure at the level of the contusion 90 min after the pulmonary contusion. Left and right upside down arrows show the chest tubes used to drain bilateral pneumothoraces. Arrows in the right and left lung show the opacification directly caused by contusion and “contre-coup”, respectively

Figure 2 shows a representative CT image illustrating sequelae of chest trauma after 90 min. Pneumothoraces were diagnosed in all animals undergoing CT.

### Changes occurring within 90 min after blunt chest trauma

Thirty minutes after contusion, all pigs except one, developed mild to moderate ARDS (PaO_2_/F_I_O_2_ 218.4 ± 73 mmHg) [45].

**Fig. 3:**
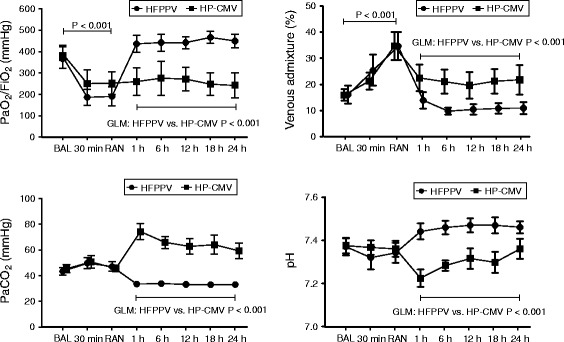
Gas exchange. Effects of high frequency inverse ratio pressure controlled ventilation (HFPPV) and moderately low V_T_ and high PEEP ventilation (HP-CMV) on the ratio of arterial partial pressure of oxygen to fraction of inspired oxygen ratio (PaO_2_/FiO_2_), venous admixture, arterial partial pressure of carbon dioxide (PaCO_2_), and pH. Data are shown as mean and standard deviation. For General Linear Model (GLM) statistics, logarithmic transformation was used for venous admixture and PaCO_2_

**Fig. 4:**
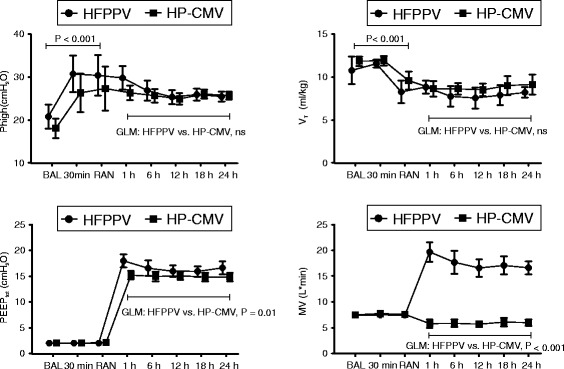
Lung mechanics. Changes of inflating pressure (P_high_), total positive end-expiratory pressure (PEEP_tot_), tidal volume (V_T_), minute ventilation (MV) for the high frequency inverse ration pressure controlled ventilation (HFPPV) and low tidal volume high PEEP ventilation (HP-CMV) groups. Data are shown as mean and standard deviation. For General Linear Model (GLM) analysis of group effects logarithmic transformation of minute ventilation was used. ns: not significant (*P* > 0.05)

**Fig. 5:**
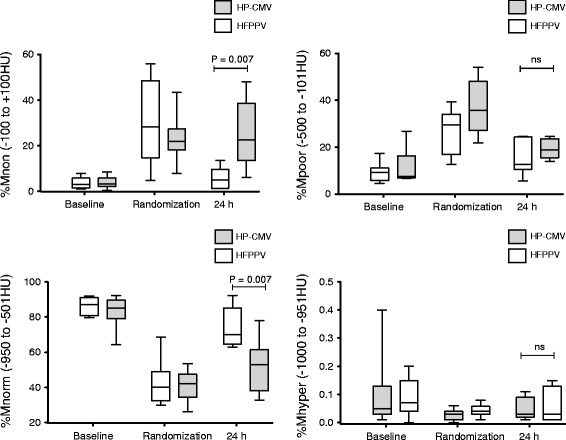
CT analysis. Effects of high frequency inverse ration pressure controlled ventilation (HFPPV) and low tidal volume high PEEP ventilation (HP-CMV) groups on percentage of: nonaerated lung (%Mnon), poorly aerated lung (%Mpoor), normally aerated lung (%Mnorm), and hyperinflated lung (%Mhyper). All CT-parameters were calculated as percentage of the total mass of tissue present in the single CT-slice. ns: not significant (*P* > 0.05)

When comparing measurements at Randomization (just before randomized group allocation) with Baseline (Figs. 3 and 4, Table 1), we observed significant decreases in PaO_2_/F_I_O_2_ (Baseline 379.9 ± 75.7 mmHg, Randomization 215.3 ± 77.7 mmHg), cardiac output (Baseline 7.3 ± 1.7 L · min^−1^, Randomization 6.2 ± 1.7 L · min^−1^), V_T_ (Baseline 11.3 ± 2.3 ml · kg^−1^, Randomization 9.0 ± 2.2 ml · kg^−1^), and heart rate (Baseline 124 ± 12 min^−1^, Randomization 107 ± 20 min^−1^), and a significant increase of Q_VA_/Q_T_ (Baseline 16.0 ± 5.3 %, Randomization 34.3 ± 9.9 %), P_high_ (Baseline 20.5 ± 4.6 cmH_2_O, Randomization 30.1 ± 8.7cmH_2_O), and mean pulmonary artery pressure (Baseline 27.5 ± 5.0 mmHg, Randomization 31.8 ± 4.9 mmHg) (*P*-values < 0.001). No statistically significant differences were observed for pulmonary capillary wedge pressure, central venous pressure, mean arterial pressure, PaCO_2_, pH, minute ventilation and PEEP (Table 1). The chest trauma caused significant increases in nonaerated (Baseline 3.7 ± 2.4 %, Randomization 25.4 ± 14.0 %) and poorly aerated lung (Baseline 10.8 ± 6.0 %, Randomization 32.3 ± 10.7 %), and a reduction in normally aerated lung (Baseline 84.0 ± 7.1 %, Randomization 41.6 ± 9.9 %,) within 90 min (*P*-values < 0.01, Fig. 5).

**Table 1:** Respiratory and hemodynamic parameters

	Group	BAL	RAN	1 h	6 h	12 h	18 h	24 h	Group effect
RR (breaths*min-1)	HFPPV	20 ± 0	20 ± 0^ns^	69.6 ± 11.7	65.6 ± 12.0	64.0 ± 13.2	62.4 ± 14.3	64.2 ± 14.2	*P* < 0.01
	HP-CMV	20 ± 0	20 ± 0^ns^	21 ± 1.8	21 ± 2.3	20.7 ± 1.8	20.7 ± 1.8	20.4 ± 1.3	
ΔP (cmH2O)	HFPPV	18.8 ± 5.1	28.4 ± 8.5*	11.8 ± 3.2	9.6 ± 3.3	8.7 ± 2.8	9.3 ± 2.8	8.2 ± 2.6	ns
	HP-CMV	16.0 ± 4.0	25.2 ± 9.3*	11.1 ± 3.3	10.7 ± 3.0	9.8 ± 3.0	11.2 ± 3.0	10.8 ± 3.0	
MAP (mmHg)	HFPPV	83.3 ± 12.8	84.1 ± 14.4^ns^	80.8 ± 13.0	78.2 ± 10.9	81.1 ± 14.3	81.0 ± 13.2	72.6 ± 13.5	*P* = 0.04
	HP-CMV	85.5 ± 14.9	87.2 ± 14.7^ns^	83.0 ± 12.2	87.6 ± 15.3	87.1 ± 16.5	91.3 ± 16.4	85.7 ± 13.1	
MPAP (mmHg)	HFPPV	28.6 ± 5.4	32.2 ± 5.5*	32.5 ± 8.7	28.7 ± 4.2	28.0 ± 3.7	29.6 ± 5.3	28.0 ± 4.3	ns
	HP-CMV	25.7 ± 3.7	29.4 ± 4.7*	34.2 ± 5.7	33.0 ± 5.4	31.8 ± 5.0	30.1 ± 7.1	30.3 ± 8.0	
CVP (mmHg)	HFPPV	9.6 ± 4.5	9.7 ± 4.5^ns^	14.2 ± 3.4	12.8 ± 3.0	12.2 ± 3.4	12.2 ± 3.4	12.3 ± 3.8	ns
	HP-CMV	10.3 ± 4.2	11.2 ± 4.0^ns^	12.3 ± 2.5	11.4 ± 2.6	12.4 ± 3.4	11.1 ± 2.6	11.1 ± 3.0	
PCWP (mmHg)	HFPPV	15.4 ± 2.5	16.7 ± 4.7^ns^	18.8 ± 3.2	17.0 ± 2.6	17.2 ± 3.5	17.5 ± 3.9	16.3 ± 2.5	ns
	HP-CMV	15.7 ± 3.3	16.4 ± 2.6^ns^	17.8 ± 3.1	17.2 ± 3.0	17.0 ± 4.3	16.0 ± 3.8	16.0 ± 4.6	
HR (min-1)	HFPPV	125 ± 14	109 ± 21*	115 ± 17	119 ± 25	127 ± 17	128 ± 13	126 ± 26	ns
	HP-CMV	121 ± 16	104 ± 20*	124 ± 27	127 ± 17	132 ± 11	141 ± 19	137 ± 18	
CO (L*min-1)	HFPPV	8.0 ± 2.3	6.5 ± 2.5*	5.8 ± 3.8	3.9 ± 0.8	4.4 ± 1.1	4.4 ± 1.5	3.8 ± 0.9	*P* < 0.01
	HP-CMV	6.9 ± 1.4	6.0 ± 1.1*	5.5 ± 1.2	5.5 ± 2.3	5.5 ± 1.5	6.2 ± 1.0	5.3 ± 0.8	
VO_2_ (ml O2*min-1)	HFPPV	392 ± 134	341 ± 206^ns^	306 ± 90	280 ± 89	317 ± 132	289 ± 131	244 ± 100	ns
	HP-CMV	288 ± 110	244 ± 101^ns^	238 ± 81	281 ± 160	286 ± 159	301 ± 143	270 ± 97	
DO_2_ (ml O_2_*min-1)	HFPPV	1040 ± 254	833 ± 290*	653 ± 171	539 ± 110	612 ± 151	615 ± 208	529 ± 123	ns
	HP-CMV	903 ± 176	778 ± 153*	678 ± 160	699 ± 297	687 ± 217	662 ± 130	654 ± 106	

Respiratory and hemodynamic parameters observed in the high frequency inverse ration pressure controlled ventilation (HFPPV) and low tidal volume high PEEP ventilation (HP-CMV) groups. Respiratory rate (RR), driving pressure (ΔP), mean arterial pressure (MAP), mean pulmonary arterial blood pressure (MPAP), central venous pressure, pulmonary capillary wedge pressure (PCWP), heart rate (HR), cardiac output (CO), oxygen consumption and delivery VO_2_ and DO_2_, respectively). Data are shown as mean and standard deviation. BAL: baseline, RAN: randomization, ns: not significant (*P* > 0.05). The superscripts in the RAN column refer to statistical significance of the comparison to BAL; *P < 0.05. Group effect (last column) indicates the statistical significance of between-group differences detected by GLM analysis of changes after randomization

### Recruitment maneuver

The median opening pressure applied during recruitment was 65cmH_2_O (IQR 50–65). All HFPPV-animals reached the target-PaO_2_ of 400 mmHg. The mean PaO_2_ immediately after recruitment maneuvers, which were considered successful, was 605 ± 67 mmHg. The hemodynamic effects of recruitment were transient: mean arterial pressure decreased but returned to pre-recruitment levels within ten minutes (Randomizatio*n* = 84.1 ± 7.1 mmHg, recruitment = 57.8 ± 8.9 mmHg, and 10 min after recruitment = 76.6 ± 18 mmHg, *P* = 0.052).

### Effects of different ventilation strategies during the treatment period

#### Respiratory variables and gas exchange

Compared to the HP-CMV-group, a significant increase in PaO_2_/F_I_O_2_ and decrease in Q_VA_/Q_T_ resulted from recruitment and start of HFPPV-ventilation. These changes persisted throughout the study (Fig. 3). Although PaCO_2_ values did not differ significantly between groups neither at Baseline (HP-CMV 46.0 ± 5.8 mmHg, HFPPV 43.2 ± 5.4 mmHg, *P* = 0.22) nor at Randomization (HP-CMV 46.3 ± 4.8 mmHg, HFPPV 46.9 ± 6.7 mmHg, *P* = 0.81), the PaCO_2_ rose and remained significantly elevated throughout the experiment (after 1 h randomized ventilation: HP-CMV 73.9 ± 23.2 mmHg *versus* HFPPV 33.2 ± 1.8 mmHg, after 24 h: HP-CMV 59.7 ± 18.3 mmHg *versus* HFPPV 33.1 ± 2.4 mmHg, group factor *P* < 0.001). While the mean pH varied only between 7.45 and 7.47 (SD ≤ 0.06) over the randomized ventilation period in the HFPPV-group, it remained below 7.30 (SD ≤ 0.08) in all but the last measurement points for the HP-CMV animals. In fact, at the measurement points after one and six hours, 92 and 75 %, respectively, of the HP-CMV animals had a pH below 7.30. The grand means of PEEP_tot_ over all measurement points after randomization differed statistically significantly between HP-CMV and HFPPV-group (14.8 ± 1.5 *versus* 16.7 ± 2.2cmH_2_O, group factor *P* = 0.015). No statistically significant differences were observed for V_T_, and P_high_ (Fig. 4).

#### Hemodynamic variables

During the 24-h study period, the HP-CMV-group showed significantly higher cardiac output, the grand means over all measurement points after randomization were 5.9 ± 1.0 L · min^−1^ for HP-CMV *versus* 4.3 ± 1.1 L · min^−1^ for HFPPV, group factor *P* = 0.009). The grand mean for arterial pressure was also higher in the HP-CMV (87.0 ± 14.7 mmHg) than in HFPPV-group 79.0 ± 13.1 mmHg, group factor *P* = 0.036). No statistically significant differences were observed for pulmonary capillary wedge pressure, central venous pressure, DO_2_, VO_2_, heart rate, mean pulmonary artery pressure (Table 1).

#### Quantitative CT analysis

At 24-h, the HFPPV-group had significantly reduced nonaerated lung (5.7 ± 4.8 % *versus* 25.2 ± 14.4 %, *P* = 0.007) and increased normally aerated lung compared to the HP-CMV-group (64.0 ± 2.4 % *versus* 41.7 ± 2.2 %, *P* = 0.007). No significant differences were found for hyperinflated and poorly aerated lung (Fig. 5).

#### Histological analysis

Histological features of lung damage are detailed in Table 2. In left dorsal lung areas, the HP-CMV-group showed significantly higher scores for atelectasis (median values: 2.5 *versus* 0, *P* = 0.01) and accumulation of inflammatory cells (median values: 1.0 *versus* 0, *P* = 0.03) compared with the HFPPV-group. Tissue samples from dorsal, gravity-dependent regions of the lung were sometimes fully atelectatic and congested and became crumbly after fixation. Therefore, some tissue samples could either not be cut or not be analyzed by microscopy. The minimum number of samples available for the dorsal, gravity-dependent lung regions was 9 for the HFPPV and 11 for the HP-CMV group.

**Table 2:** Histological analysis

		HFPPV group		HP-CMV group		Group effect	
		Right	Left	Right	Left	Right	Left
Atelectasis	Non-dependent	0 (0–0)	0 (0–0)	0 (0–0)	0 (0–3)	ns	ns
	Central	0 (0–1)	0 (0–3)	0 (0–1)	0 (0–3)	ns	ns
	Dependent	0 (0–3)	0 (0–3)	2 (0–3)	2.5 (1–3)	ns	*P* = 0.01
Edema	Non-dependent	0 (0–2)	0 (0–3)	0 (0–1)	0 (0–1)	ns	ns
	Central	0 (0–3)	0 (0–3)	0 (0–1)	0 (0–2)	ns	ns
	Dependent	0 (0–1)	0 (0–1)	0 (0–1)	0 (0–1)	ns	ns
Inflammation	Non-dependent	0 (0–0)	0 (0–0)	0 (0–2)	0 (0–0)	ns	ns
	Central	0 (0–0)	0 (0–2)	0 (0–1)	0 (0–3)	ns	ns
	Dependent	0 (0–1)	0 (0–0)	0 (0–3)	1 (0–3)	ns	*P* = 0.03
Hemorrhage	Non-dependent	0 (0–2)	0 (0–1)	0 (0–0)	0 (0–1)	ns	ns
	Central	0 (0–1)	0 (0–2)	0 (0–3)	1 (0–2)	ns	ns
	Dependent	1 (0–2)	1 (0–2)	1 (0–2)	1 (0–2)	ns	ns

Histological evaluation of parenchymal damage in different lung zones of pigs in the high frequency inverse ration pressure controlled ventilation (HFPPV) and low tidal volume high PEEP ventilation (HP-CMV) groups: non-dependent (ventral), central and dependent (dorsal). Values are shown as median (minimum - maximum). The maximum score achievable was three for all parameters. Inflammation refers to accumulation of inflammatory cells in airspaces and interstitium. ns: not significant, *P* > 0.05

## Discussion

Our results demonstrate that, over an extended experimental period of 24 h, both strategies for mechanical ventilation, HFPPV and HP-CMV, enabled the use of moderately low tidal volumes and limited inflating pressures in pigs with mild ARDS after blunt chest trauma. Contrasting with the approach to conventional mechanical ventilation used here, however, improvements in lung function, e.g. oxygenation, CO_2_ elimination and lung aeration were detected only in the HFPPV-group.

Individuals developing ARDS after trauma and in particular blunt chest trauma have been significantly underrepresented in clinical and experimental studies on mechanical ventilation and thus controversy remains about how to ventilate these patients, who are at high risk of developing ARDS or ventilator-associated lung injury, if ventilators settings are inappropriately chosen [10, 13–16, 46].

The rationales of our ventilation strategies differ significantly and although the results may appear somewhat predestined by the design of the study, the strategies chosen represent the way mechanical ventilation is employed by clinicians. The ventilation strategy adopted here for the HP-CMV-group inherently tolerates persisting or even progressive lung collapse, which has been associated with hypercapnia, acidosis, surfactant loss and/or dysfunction and development of edema and hyperinflation of the lung that remains aerated [47–51]. We tried to minimize this effect by choosing a rather high PEEP in the HP-CMV-group. This PEEP is compatible with previous studies, but is still higher than the PEEP currently used in clinical practice for mild-to-moderate ARDS [1, 2, 39, 45]. Although they are obvious confounders of the results observed, the limits chosen for V_T_ and respiratory rate also reflect current clinical considerations for the implementation of lung protective mechanical ventilation and thus were intentionally chosen for testing our hypothesis [2, 52–54]. The Open Lung Concept in contrast aims at actively recruiting nonaerated lung as early as possible. This is expected to lower alveolar opening pressures and homogenize lung aeration, resulting in reduced parenchymal stress during tidal ventilation [19, 37, 55, 56]. Individualized PEEP is applied to stabilize the resulting gains in lung aeration and function, which are otherwise short lived, until recovery of lung function, surfactant system and parenchymal injury [19, 37, 48, 56, 57].

The general improvement of lung function in our HFPPV-group, which was paralleled by subgroup-results of quantitative CT analysis and histology, can be interpreted in support of the Open Lung Concept for lung protective ventilation (Tables 1 and 2, Figs. 3 and 5). Although statistically significant, the between-group difference in the grand means for PEEP_tot_ was only 1.8cmH_2_O and thus our findings seem to reflect much more recruitment and/or minute ventilation effects than differences in PEEP. It appears interesting in this context that the results in HFPPV developed despite a randomization bias with an apparently more severe injury in the HFPPV group (Figs. 3 and 4).

The HFPPV resulted in significantly better CO_2_-elimination compared to the conventional ventilation approach chosen for our HP-CMV-group, where PaCO_2_ rose and pH decreased to levels that may be unacceptable in trauma patients with concomitant brain trauma and/or multifactorial impairments of coagulation (Fig. 3) [11–13, 47, 58]. After one and six hours of HP-CMV ventilation, 92 and 75 %, respectively, of all pigs in this group had a pH below 7.30. This better PaCO_2_-control during HFPPV-ventilation may be of particular interest in trauma patients. However, although mild hypocapnia as observed here as well as in our earlier clinical case series may help managing acute increases in intracranial pressure, it should only be used under close monitoring to exclude cerebral tissue hypoxia [6]. If necessary, PaCO_2_ can be increased to normocapnia simply by increasing apparatus dead-space [59].

The decrease of 50 % in PaCO_2_ between HFPPV and our HP-CMV-group is less than what should be theoretically expected from the higher respiratory rate. The PaCO_2_ is affected by four main factors: MV, V_T_, dead-space (VD) and CO_2_-production ($\overset{\bullet }{V}C{O}_2$), according to the equation $PaC{O}_2=K\cdot \overset{\bullet }{V}C{O}_2/\left(MV\cdot \left(1-VD\;/\;VT\right)\right)$, where K is a constant [60]. Since MV increased about threefold from HP-CMV to HFPPV, theoretically PaCO_2_ should decrease roughly 70 % from HP-CMV to HFPPV, and not only 50 %. Thus other factors of the equation must differ between groups. Since V_T_ did not change significantly, and considering that there is no obvious reason for a higher CO_2_-production in the HFPPV-group, a larger anatomical dead-space, e.g. by recruitment and/or inflation of airways, is the most likely explanation.

Although the high respiratory rate facilitated V_T_ reduction, it should be noticed that the higher respiratory rate in the HFPPV-group resulted in higher mechanical work performed by the ventilator, and consequently larger energy transferred to and dissipated by the respiratory system. In PCV, the work performed by the ventilator per minute is *W* = *P*_*high*_ ⋅ *V*_*T*_ ⋅ *respiratory rate*. Because V_T_ and P_high_ did not differ significantly, the work per minute in HFPPV was about 3.25 times that in HP-CMV, since this is the ratio between the respiratory rates in the two groups. However, the relevance of this difference in terms of potential of injury is far from being established, and our histological analysis did not indicate injurious effects of the HFPPV.

Although the HFPPV may help achieving lung protective ventilation, an important concern should be the avoidance of potential pulmonary and hemodynamic side effects [59, 61]. Already the mechanical ventilation with high PEEP, as in both groups, requires increased awareness of the potential development of pneumothorax, especially after chest trauma. The same applies to recruitment, which was therefore performed for only 10 s and a target-PaO_2_ above 400 mmHg was used as individual indicator for sufficient recruitment [40, 41]. Because the P_high_ for recruitment was applied during HFPPV, the pressure at the distal (tracheal) end of the endotracheal tube will be considerably lower. Especially during high inspiratory flows, this pressure drop can reach 7cmH_2_O. Also, already after the first breath, high PEEP_tot_ develops due to the development of PEEP_int_, which further reduces the pressure amplitude. Nevertheless, all our pigs had chest tubes in place and thus pneumothoraces, which may have develop after recruitment or during ventilation with high PEEP in both groups, may have gone undetected. Although we never observed large air leaks, which should have resulted if gross lung rupture had occurred, our data do not provide any proof of safety of recruitment. Individuals without chest tubes, in whom recruitment or ventilation with high PEEP is performed, should be closely monitored for complications.

The smaller values for some hemodynamic parameters measured in the HFPPV-group did not reflect in any way a clinically unacceptable impairment in hemodynamics. Mean arterial pressure decreased transiently after recruitment and could be easily managed by injection of small doses of norepinephrine and returned to pre-recruitment levels within ten minutes. Also, the higher values of mean arterial pressure and cardiac output observed in the HP-CMV-group may well be explained by permissive hypercapnia [3]. Interestingly, the mean pulmonary artery pressure as a surrogate of right heart afterload, whose limitation is a therapeutic problem in ARDS tended to be lower during HFPPV, likely reflecting released hypoxic pulmonary vasoconstriction and higher lung volumes [59].

## Limitations

The blunt chest trauma model used here is affected by uncontrolled factors (e.g. angle and rebound of the firing device, energy density transferred to the subject, and undesired extra-pulmonary injuries), which limit the between- and within-study comparability as illustrated by the randomization bias visible for PaO_2_/FiO_2_ ratio and P_high_ in Figs. 3 and 4, respectively. There was a significant drop-out of animals due to the trauma. The injury was obviously not confined to the ipsilateral lung, but (as often in clinical reality) involved the contralateral lung and other organs. Although the drop-out cases complicate the interpretation, they were equally distributed and thus did not skew the results. However, we are currently modifying the chest trauma model to eliminate the drawbacks mentioned. Single-slice CT focusing on the morphological assessment of the contusion was performed only in a subgroup of animals and only for three time points, which limits the impact of this data. Besides changes over time, cranio-caudal differences in lung aeration might have gone undetected. Also, quantitative CT analysis did not reveal relevant hyperinflation in any group. Besides an absence of hyperinflation *per se*, this may be explained by general problems related to quantitative CT analysis. Our histology analysis is only basic and we cannot provide biochemical results characterizing pulmonary inflammation. All animals developed pneumothoraces after chest trauma. Because the insertion of chest tubes compromises the assessment of lung mechanics, we present only basic lung mechanics. Expiration was deliberately terminated in HFPPV animals in order to generate intrinsic PEEP. Because of this common dead-space analysis using volumetric capnography could not be used. We acknowledge the importance of information regarding dead-space and alveolar ventilation and we will explore alternative methods for assessing dead-space and alveolar ventilation in further experiments. Finally, although the present experiment confirmed our earlier clinical experience, extrapolation from the porcine model to the clinical scenario requires caution, not only because the effects of HFPPV may depend on body position and muscular tone.

## Conclusions

Conventional mechanical ventilation and therapeutic adjuncts in ARDS may be challenged in trauma patients because of instable fractures, severe brain injury, or impaired coagulation. Using a porcine model mimicking ARDS due to blunt chest trauma, we demonstrated that HFPPV ventilation involving lung recruitment and high total PEEP improved oxygenation and lung aeration while avoiding CO_2_-accumulation and acidosis, which are particularly useful effects in patients with posttraumatic ARDS. The HFPPV offers an option to avoid (too) permissive impairments in lung aeration and gas exchange during lung protective mechanical ventilation and may be tested before employing more invasive modalities such as extracorporeal CO_2_-removal.

## Abbreviations

$$ \overset{\bullet }{V}C{O}_2 $$: CO_2_-production
ARDS: acute respiratory distress syndrome
DO_2_: Oxygen delivery
F_I_O_2_: fraction of inspired oxygen
HFPPV: high-frequency inverse-ratio pressure-controlled ventilation
HP-CMV: conventional mechanical ventilation with moderately low V_T_ and high PEEP_ext_
PCV: pressure-controlled ventilation
PEEP: positive end-expiratory pressure
PEEP_ext_: external PEEP
PEEP_int_: intrinsic PEEP
PEEP_tot_: total PEEP
P_high_: inflating pressure
Q_VA_/Q_T_: venous admixture
VD: dead-space
VO_2_: oxygen consumption
V_T_: tidal volume

## References

[1] Amato MB, Barbas CS, Medeiros DM, Magaldi RB, Schettino GP, Lorenzi-Filho G (1998). Effect of a protective-ventilation strategy on mortality in the acute respiratory distress syndrome. N Engl J Med.

[2] Brower RG, Lanken PN, MacIntyre N, Matthay MA, Morris A, Ancukiewicz M (2004). Higher *versus* lower positive end-expiratory pressures in patients with the acute respiratory distress syndrome. N Engl J Med.

[3] Carvalho CR, Barbas CS, Medeiros DM, Magaldi RB, Lorenzi Filho G, Kairalla RA (1997). Temporal hemodynamic effects of permissive hypercapnia associated with ideal PEEP in ARDS. Am J Respir Crit Care Med.

[4] Jeremitsky E, Omert L, Dunham CM, Protetch J, Rodriguez A (2003). Harbingers of poor outcome the day after severe brain injury: hypothermia, hypoxia, and hypoperfusion. J Trauma.

[5] Johannigman JA, Miller SL, Davis BR, Davis K, Campbell RS, Branson RD (2003). Influence of low tidal volumes on gas exchange in acute respiratory distress syndrome and the role of recruitment maneuvers. J Trauma.

[6] Schreiter D, Reske A, Stichert B, Seiwerts M, Bohm SH, Kloeppel R (2004). Alveolar recruitment in combination with sufficient positive end-expiratory pressure increases oxygenation and lung aeration in patients with severe chest trauma. Crit Care Med.

[7] McKinley BA, Kozar RA, Cocanour CS, Valdivia A, Sailors RM, Ware DN (2002). Normal *versus* supranormal oxygen delivery goals in shock resuscitation: the response is the same. J Trauma.

[8] Bein T, Reber A, Metz C, Jauch KW, Hedenstierna G (1998). Acute effects of continuous rotational therapy on ventilation-perfusion inequality in lung injury. Intensive Care Med.

[9] Davis K, Johannigman JA, Campbell RS, Marraccini A, Luchette FA, Frame SB (2001). The acute effects of body position strategies and respiratory therapy in paralyzed patients with acute lung injury. Crit Care.

[10] Putensen C, Zech S, Wrigge H, Zinserling J, Stüber F, Von Spiegel T (2001). Long-term effects of spontaneous breathing during ventilatory support in patients with acute lung injury. Am J Respir Crit Care Med.

[11] Forster N, Engelhard K (2004). Managing elevated intracranial pressure. Curr Opin Anaesthesiol.

[12] Davenport R (2013). Pathogenesis of acute traumatic coagulopathy. Transfusion.

[13] Mascia L, Zavala E, Bosma K, Pasero D, Decaroli D, Andrews P (2007). High tidal volume is associated with the development of acute lung injury after severe brain injury: an international observational study. Crit Care Med.

[14] Holland MC, Mackersie RC, Morabito D, Campbell AR, Kivett VA, Patel R (2003). The development of acute lung injury is associated with worse neurologic outcome in patients with severe traumatic brain injury. J Trauma.

[15] Salim A, Martin M, Brown C, Inaba K, Browder T, Rhee P (2008). The presence of the adult respiratory distress syndrome does not worsen mortality or discharge disability in blunt trauma patients with severe traumatic brain injury. Injury.

[16] Maxwell RA, Green JM, Waldrop J, Dart BW, Smith PW, Brooks D (2010). A randomized prospective trial of airway pressure release ventilation and low tidal volume ventilation in adult trauma patients with acute respiratory failure. J Trauma.

[17] Andrews PL, Shiber JR, Jaruga-Killeen E, Roy S, Sadowitz B, O’Toole RV (2013). Early application of airway pressure release ventilation may reduce mortality in high-risk trauma patients: A systematic review of observational trauma ARDS literature. J Trauma Acute Care Surg.

[18] Probst C, Pape H-C, Hildebrand F, Regel G, Mahlke L, Giannoudis P (2009). 30 years of polytrauma care: An analysis of the change in strategies and results of 4849 cases treated at a single institution. Injury.

[19] Lachmann B, Jonson B, Lindroth M, Robertson B (1982). Modes of artificial ventilation in severe respiratory distress syndrome. Lung function and morphology in rabbits after wash-out of alveolar surfactant. Crit Care Med.

[20] Lachmann B (1992). Open up the lung and keep the lung open. Intensive Care Med.

[21] Papadakos PJ, Lachmann B (2007). The Open Lung Concept of Mechanical Ventilation: The Role of Recruitment and Stabilization. Crit Care Clin.

[22] Reske A, Seiwerts M, Reske A, Gottschaldt U, Schreiter D (2006). Early recovery from post-traumatic acute respiratory distress syndrome. Clin Physiol Funct Imaging.

[23] Sjöstrand UH, Lichtwarck-Aschoff M, Nielsen JB, Markström A, Larsson A, Svensson BA (1995). Different ventilatory approaches to keep the lung open. Intensive Care Med.

[24] Dongelmans DA, Paulus F, Veelo DP, Binnekade JM, Vroom MB, Schultz MJ (2011). Adaptive support ventilation may deliver unwanted respiratory rate-tidal volume combinations in patients with acute lung injury ventilated according to an open lung concept. Anesthesiology.

[25] Habashi NM (2005). Other approaches to open-lung ventilation: airway pressure release ventilation. Crit Care Med.

[26] Mercat A, Titiriga M, Anguel N, Richard C, Teboul JL (1997). Inverse ratio ventilation (I/E = 2/1) in acute respiratory distress syndrome: a six-hour controlled study. Am J Respir Crit Care Med.

[27] Whitwam JG (1984). Functional dead space during high frequency ventilation. Eur J Anaesthesiol.

[28] Syring RS, Otto CM, Spivack RE, Markstaller K, Baumgardner JE (2007). Maintenance of end-expiratory recruitment with increased respiratory rate after saline-lavage lung injury. J Appl Physiol.

[29] Institute of Laboratory Animal Resources C on LS: No Title. *Inst Lab Anim Resour Comm Life Sci Natl Res Counc* http://www.nap.edu/read/5140/chapter/1*] Natl Acad Press Washington, DC* 2004.

[30] Pesenti A, Riboni A, Marcolin R, Gattinoni L (1983). Venous admixture (Qva/Q) and true shunt (Qs/Qt) in ARF patients: effects of PEEP at constant FIO2. Intensive Care Med.

[31] Hellinger A, Konerding MA, Malkusch W, Obertacke U, Redl H, Bruch J (1995). Does lung contusion affect both the traumatized and the noninjured lung parenchyma? A morphological and morphometric study in the pig. J Trauma.

[32] Obertacke U, Neudeck F, Majetschak M, Hellinger A, Kleinschmidt C, Schade FU (1998). Local and systemic reactions after lung contusion: an experimental study in the pig. Shock.

[33] Cohn SM, Zieg PM (1996). Experimental pulmonary contusion: review of the literature and description of a new porcine model. J Trauma.

[34] Melton SM, Davis KA, Moomey CB, Fabian TC, Proctor KG (1999). Mediator-dependent secondary injury after unilateral blunt thoracic trauma. Shock.

[35] Rothen HU, Sporre B, Engberg G, Wegenius G, Reber A, Hedenstierna G (1995). Prevention of atelectasis during general anaesthesia. Lancet.

[36] Vieira SR, Puybasset L, Lu Q, Richecoeur J, Cluzel P, Coriat P (1999). A scanographic assessment of pulmonary morphology in acute lung injury. Significance of the lower inflection point detected on the lung pressure-volume curve. Am J Respir Crit Care Med.

[37] Suarez-Sipmann F, Böhm SH, Tusman G, Pesch T, Thamm O, Reissmann H (2007). Use of dynamic compliance for open lung positive end-expiratory pressure titration in an experimental study. Crit Care Med.

[38] Amato MB, Barbas CS, Medeiros DM, Schettino GDP, Lorenzi Filho G, Kairalla RA (1995). Beneficial effects of the “open lung approach” with low distending pressures in acute respiratory distress syndrome. A prospective randomized study on mechanical ventilation. Am J Respir Crit Care Med.

[39] Villar J, Kacmarek RM, Pérez-Méndez L, Aguirre-Jaime A (2006). A high positive end-expiratory pressure, low tidal volume ventilatory strategy improves outcome in persistent acute respiratory distress syndrome: a randomized, controlled trial. Crit Care Med.

[40] Borges JB, Okamoto VN, Matos GFJ, Caramez MPR, Arantes PR, Barros F (2006). Reversibility of lung collapse and hypoxemia in early acute respiratory distress syndrome. Am J Respir Crit Care Med.

[41] Reske AW, EL Costa V, Reske AP, Rau A, Borges JB, Beraldo MA (2013). Bedside estimation of nonaerated lung tissue using blood gas analysis. Crit Care Med.

[42] Reske AW, Busse H, Amato MBP, Jaekel M, Kahn T, Schwarzkopf P (2008). Image reconstruction affects computer tomographic assessment of lung hyperinflation. Intensive Care Med.

[43] Spieth PM, Knels L, Kasper M, Domingues Quelhas A, Wiedemann B, Lupp A (2007). Effects of vaporized perfluorohexane and partial liquid ventilation on regional distribution of alveolar damage in experimental lung injury. Intensive Care Med.

[44] Quintel M, Heine M, Hirschl RB, Tillmanns R, Wessendorf V (1998). Effects of partial liquid ventilation on lung injury in a model of acute respiratory failure: a histologic and morphometric analysis. Crit Care Med.

[45] Ranieri VM, Rubenfeld GD, Thompson BT, Ferguson ND, Caldwell E, Fan E (2012). Acute respiratory distress syndrome: the Berlin Definition. JAMA.

[46] Gajic O, Frutos-Vivar F, Esteban A, Hubmayr RD, Anzueto A (2005). Ventilator settings as a risk factor for acute respiratory distress syndrome in mechanically ventilated patients. Intensive Care Med.

[47] Batchinsky AI, Jordan BS, Regn D, Necsoiu C, Federspiel WJ, Morris MJ (2011). Respiratory dialysis: reduction in dependence on mechanical ventilation by venovenous extracorporeal CO2 removal. Crit Care Med.

[48] Aufmkolk M, Fischer R, Voggenreiter G, Kleinschmidt C, Schmit-Neuerburg KP, Obertacke U (1999). Local effect of lung contusion on lung surfactant composition in multiple trauma patients. Crit Care Med.

[49] Raghavendran K, Davidson BA, Hutson AD, Helinski JD, Nodzo SR, Notter RH (2009). Predictive modeling and inflammatory biomarkers in rats with lung contusion and gastric aspiration. J Trauma.

[50] Van Kaam AH, Lachmann RA, Herting E, De Jaegere A, van Iwaarden F, Noorduyn LA (2004). Reducing atelectasis attenuates bacterial growth and translocation in experimental pneumonia. Am J Respir Crit Care Med.

[51] Terragni PP, Rosboch G, Tealdi A, Corno E, Menaldo E, Davini O (2007). Tidal hyperinflation during low tidal volume ventilation in acute respiratory distress syndrome. Am J Respir Crit Care Med.

[52] Patroniti N, Pesenti A (2003). Low tidal volume, high respiratory rate and auto-PEEP: the importance of the basics. Crit Care.

[53] Vieillard-Baron A, Prin S, Augarde R, Desfonds P, Page B, Beauchet A (2002). Increasing respiratory rate to improve CO2 clearance during mechanical ventilation is not a panacea in acute respiratory failure. Crit Care Med.

[54] De Durante G, Del Turco M, Rustichini L, Cosimini P, Giunta F, Hudson LD (2002). ARDSNet lower tidal volume ventilatory strategy may generate intrinsic positive end-expiratory pressure in patients with acute respiratory distress syndrome. Am J Respir Crit Care Med.

[55] Gattinoni L, Pesenti A (2005). The concept of “baby lung”. Intensive Care Med.

[56] Koh W-J, Suh GY, Han J, Lee S-H, Kang EH, Chung MP (2005). Recruitment maneuvers attenuate repeated derecruitment-associated lung injury. Crit Care Med.

[57] McCarthy MC, Cline AL, Lemmon GW, Peoples JB (1999). Pressure control inverse ratio ventilation in the treatment of adult respiratory distress syndrome in patients with blunt chest trauma. Am Surg.

[58] Davis DP (2008). Early ventilation in traumatic brain injury. Resuscitation.

[59] Mekontso Dessap A, Charron C, Devaquet J, Aboab J, Jardin F, Brochard L (2009). Impact of acute hypercapnia and augmented positive end-expiratory pressure on right ventricle function in severe acute respiratory distress syndrome. Intensive Care Med.

[60] Wexler HR, Lok P (1981). A simple formula for adjusting arterial carbon dioxide tension. Can Anaesth Soc J.

[61] Fan E, Wilcox ME, Brower RG, Stewart TE, Mehta S, Lapinsky SE (2008). Recruitment maneuvers for acute lung injury: a systematic review. Am J Respir Crit Care Med.

